# Expression of Genes for Drug Transporters in the Human Female Genital Tract and Modulatory Effect of Antiretroviral Drugs

**DOI:** 10.1371/journal.pone.0131405

**Published:** 2015-06-23

**Authors:** Karolin Hijazi, Anna M. Cuppone, Kieron Smith, Maria A. Stincarelli, Julia Ekeruche-Makinde, Giulia De Falco, Georgina L. Hold, Robin Shattock, Charles G. Kelly, Gianni Pozzi, Francesco Iannelli

**Affiliations:** 1 School of Medicine & Dentistry, University of Aberdeen, Aberdeen, Scotland, United Kingdom; 2 Laboratory of Molecular Microbiology and Biotechnology, Department of Medical Biotechnologies, University of Siena, Siena, Italy; 3 MICROBIOTEC srl, Siena, Italy; 4 Mucosal Infection & Immunity Group, Section of Infectious Diseases, Imperial College, London, United Kingdom; 5 Dental Institute, King’s College London, London, United Kingdom; Commissariat a l'Energie Atomique(cea), FRANCE

## Abstract

Anti-retroviral (ARV) –based microbicides are one of the strategies pursued to prevent HIV-1 transmission. Delivery of ARV drugs to subepithelial CD4+ T cells at concentrations for protection is likely determined by drug transporters expressed in the cervicovaginal epithelium. To define the role of drug transporters in mucosal disposition of topically applied ARV-based microbicides, these must be tested in epithelial cell line-based biopharmaceutical assays factoring the effect of relevant drug transporters. We have characterised gene expression of influx and efflux drug transporters in a panel of cervicovaginal cell lines and compared this to expression in cervicovaginal tissue. We also investigated the effect of dapivirine, darunavir and tenofovir, currently at advanced stages of microbicides development, on expression of drug transporters in cell lines. Expression of efflux ABC transporters in cervical tissue was best represented in HeLa, Ect1/E6E7 and End1/E6E7 cell lines. Expression of influx OCT and ENT transporters in ectocervix matched expression in Hela while expression of influx SLCO transporters in vagina was best reflected in VK2/E6E7 cell line. Stimulation with darunavir and dapivirine upregulated MRP transporters, including MRP5 involved in transport of tenofovir. Dapivirine also significantly downregulated tenofovir substrate MRP4 in cervical cell lines. Treatment with darunavir and dapivirine showed no significant effect on expression of BCRP, MRP2 and P-glycoprotein implicated in efflux of different ARV drugs. Darunavir strongly induced expression in most cell lines of CNT3 involved in cell uptake of nucleotide/nucleoside analogue reverse transcriptase inhibitors and SLCO drug transporters involved in cell uptake of protease inhibitors. This study provides insight into the suitability of cervicovaginal cell lines for assessment of ARV drugs in transport kinetics studies. The modulatory effect of darunavir and dapivirine on expression of drug transporters involved in transport of tenofovir points to the possibility of combining these drugs to improve retention of individual drugs at target tissues.

## Introduction

Microbicides are one of the strategies pursued to prevent transmission of HIV-1. They have the potential to inhibit or block early events of HIV-1 infection when applied directly to the vaginal or rectal mucosae. While earlier generation microbicides aimed at disrupting the virus or inhibiting attachment and fusion showed no efficacy in clinical trials [1–5], the CAPRISA 004 trial of vaginally-applied tenofovir gel demonstrated 39% protection against HIV-1 infection providing significant boost for the development of anti-retroviral (ARV)-based microbicides [6]. More recent clinical trials of tenofovir gel did not confirm the protective effect shown in CAPRISA 004 and have attributed the lack of protection to lack of compliance [7,8]. Therefore, the development of improved strategies for formulation and delivery is urgently required.

Protease, integrase and reverse transcriptase inhibitors are currently in the pipeline of microbicides development [9]. To exert a protective effect, ARV-based microbicides must cross the cervicovaginal epithelium and distribute to underlying CD4+ T cells which have been confirmed as primary target cells of sexually transmitted HIV-1 particles [10]. The mucosal disposition of ARV drugs in cervicovaginal mucosae and delivery of active and sustained concentrations for protection at subepithelial CD4+ T cells is likely determined not only by physicochemical properties of the drug but also by the effects of drug uptake and efflux transporters expressed mainly in the cervicovaginal epithelium. Recently, drug transporters that may be relevant for vaginal microbicides have been described in cervicovaginal tissue [11,12]. The influence of identified drug transporters on the distribution of ARV-based microbicides at target cells of HIV-1 remains to be determined.

Among the anti-retroviral drugs that are most advanced in clinical development as microbicides, some may well cross the epithelium through passive transports mechanisms. For example polar small molecule drugs such as tenofovir (nucleotide analogue reverse transcriptase inhibitor) may cross the epithelium by paracellular routes and hydrophobic drugs such as dapivirine (non-nucleoside analogue reverse transcriptase inhibitor) may partition directly into cell membranes. On the other hand, reverse transcriptase as well as protease inhibitors have also been identified as substrates for both influx and efflux transporters. In particular, influx transporters of the SLC22 (OCT2 and OCT3), SLC28 (CNT) and SLC29 (ENT1) families as well as MRP4 and MRP5 efflux transporters that have been reported as expressed in cervicovaginal epithelium are involved in transport of several nucleoside analogue anti-retroviral drugs [13]. Equally protease inhibitors including promising candidates for microbicides development such as saquinavir and darunavir are substrates for influx transporter OATPD (member of the SLCO family) and efflux transporters P-glycoprotein (P-gp) and MRP1 [14] all reported as expressed in human ectocervix and vagina [12]. Anti-retroviral drugs can also modulate functionality and expression of drug transporters. Reverse transcriptase inhibitors affect the activity of BCRP [15] and MRP efflux transporters [16] as well as several influx transporters [14]. Similarly, protease inhibitors modulate OATP [17] and OCT influx transporters as well as P-gp, MRP and BCRP efflux transporters [14,15], although darunavir has been shown to inhibit P-gp activity while inducing expression of this transporter [18].

To further characterise the relevance of drug efflux and uptake transporters for cervicovaginal mucosal disposition of topically applied ARV-based microbicides, these must be tested in biopharmaceutical assays factoring the effect of relevant drug transporters. Cell line-based assays are useful models to enable high-throughput biopharmaceutical screening of candidate microbicides. For this purpose we have characterised gene expression of influx and efflux drug transporters in a panel of cell lines derived from human vagina, ectocervix, endocervix and endometrium. We compared drug transporters expression in cell lines to expression in cervicovaginal explants to determine the suitability of the cell lines as surrogates of cervicovaginal tissue for use in *in vitro* assays. We also investigated the effect of three ARV drugs (dapivirine, darunavir and tenofovir), currently at advanced stages of development as microbicides, on expression of drug transporters in the cervicovaginal cell lines.

## Material & Methods

### Ethics statement

Ethical approval for involvement of women in this study was granted by the Research Ethics Committees of the University of Siena and Imperial College Healthcare NHS Trust. Written informed consent was obtained from all subjects according to the World Health Organisation guidelines for good clinical practice (GCP) and the local Research Ethics Committee policies.

### Patient recruitment and cervicovaginal tissue collection

Biopsies were collected from 10 pre-menopausal female subjects (aged 43–54) undergoing planned therapeutic hysterectomy for uterine fibromatosis except for one subject affected by multiple myomas. Only tissue with no histologic evidence of neoplastic or inflammatory disease was analysed. Tissues collected at the University of Siena during surgery were snap frozen in liquid nitrogen and immediately used for RNA purification. Samples collected from St Mary’s Hospital London were frozen in a solution containing 10% Dimethyl Sulfoxide (DMSO, Sigma-Aldrich, Dorset, UK) in Foetal Bovine Serum (FBS) obtained from Life Technologies (Paisley, UK) and transported to the University of Siena for analysis.

### Cell lines, growth media and drugs

Cell lines were all obtained from American Type Culture Collection (ATCC, Manassas, VA, USA). All cell culture reagents were from Gibco (Life Technologies Italia, Monza, MB, Italy) unless otherwise stated. Cell lines were all grown for a maximum of 5 passages before cell lysis and mRNA analysis. HeLa cell line (ATCC CCL-2), derived from human cervical epithelial adenocarcinoma, was grown in Dulbecco's Modified Eagle's Medium (DMEM) supplemented with 10% FBS. VK2/E6E7 (ATCC CRL-2616), Ect1/E6E7 (ATCC CRL-2614) and End1/E6E7 (ATCC CRL-2615), obtained by immortalisation of primary vaginal/ectocervical/endocervical epithelial cells respectively [19], were grown in Keratinocyte-Serum Free Medium (KSFM) supplemented with 0.1 ng/ml human recombinant Epidermal Growth Factor (EGF), 0.05 mg/ml Bovine Pituitary Extract and 0.4 mM CaCl_2_. HEC-1A cell line (ATCC HTB-112), derived from human endometrial epithelial adenocarcinoma, was grown in McCoy’s 5A Modified Medium supplemented with 10% FBS. Culture media were supplemented with 100 U/ml penicillin and 100 μg/ml streptomycin (Sigma-Aldrich, Milan, Italy). Dapivirine was purchased from Selleckchem (Suffolk, UK). Darunavir and tenofovir were kindly provided by Janssen R&D Ireland (Cork, Ireland) and Gilead Sciences (Foster City, CA, USA) respectively. A 100 mM stock solution of dapivirine (MW 329.4) and a 250 mM stock solution of darunavir (MW 547.6) was prepared in DMSO and stored at -70°C. A 10 mM stock solution of tenofovir (MW 287.2) was freshly prepared in cell culture media.

### Cell viability assay

Cell line monolayers were washed with sterile PBS and harvested using trypsin/EDTA. Trypsin/EDTA was neutralized with a washing step in DMEM/10% FBS. Cells were seeded at 5 × 10^4^ cell/well in fresh medium in a 96-well plate and incubated at 37°C for 12 hours. Medium was then removed and cells were washed twice with sterile PBS before treatment with drugs. Drug stimuli were added at serial dilutions at the following ranges of concentrations: 0–100 μM Dapivirine, 0–2000 μM Darunavir, 0–1000 μM Tenofovir. Three technical replicates were prepared for each experiment. Cell viability was measured at 24, 72 and 168 hours after drug treatment using the TACS XTT Cell Proliferation Assay Kit (Trevigen, Gaithersburg, USA) according to manufacturer's instructions. Briefly, stimuli were removed and cells were washed twice with sterile PBS, 100 μl of the appropriate medium without red phenol and serum plus 50 μl of XTT working solution were added to each well. The plates were incubated at 37°C for 2 hours and the optical density was measured using a 620 nm reference filter. Cell viability was calculated as the percent change in absorbance relative to untreated cells.

### Cell line stimulation with drugs

Cells were seeded at 1,5x10^6^ in appropriate media in a 12 cm^2^ tissue culture flask and incubated at 37°C for 12 hours. Drug stimuli were added at the following concentrations showing no cytotoxicity in XTT assays and maintaining solubility in culture medium: 10 μM dapivirine, 250 μM darunavir, 1000 μM tenofovir and 0.1% DMSO as control where appropriate. Cells were stimulated for 24,72 and 168 hours, harvested by centrifugation at 200 g for 10 minutes at 4°C, resuspended in 1 ml of RNAlater Solution (Ambion, Life Technologies) and then stored at -80°C until total RNA extraction. Incubation period was in line with length of stimulation used in previous studies [20]. All cell line stimulation experiments were conducted as a minimum of three biological repeats.

### Purification of total RNA, cDNA synthesis and quantitative PCR

Total RNA was extracted from cell lines and human tissues using the NucleoSpin RNA II Isolation Kit (Macherey-Nagel, Germany) according manufacturer's instructions. Cells were thawed and centrifuged at 200 g for 10 minutes at 4°C following addition of 1 ml of cold sterile PBS. Pellets were suspended in RA1 Lysis Buffer containing guanidium thiocynate and 20 mM DTT (1,4-dithiothreitol) and mixed vigorously. Biopsies were homogenized in RA1 Lysis Buffer using 5 mm stainless steel beads in the Tissues Lyser apparatus (Qiagen s.r.l., Milan, Italy). RNA samples were treated with DNase on column. Integrity and quantification of RNA were evaluated using the Agilent RNA 6000 Nano Kit (Agilent Technologies, Milan, Italy) on the Agilent 2100 Bioanalyser (Agilent Technologies). RNA samples were stored at -80°C until reverse transcription.

Complementary DNA synthesis was performed from total RNA using the QuantiTect Reverse Transcription Kit (Qiagen) following the protocol supplied by the manufacturer. Reverse transcription experiments were carried out at 42°C for 30 minutes using 1 μg of total RNA as starting template and the RT random primers mix in presence of RNase inhibitor. Complementary DNA samples were stored at -20°C until quantitative PCR.

Quantitative PCR was performed using the TaqMan PCR Array Human Drug Transporters Fast 96-well system and the TaqMan Fast Advanced Master Mix (Life Technologies). The PCR array is pre-configured with lyophilised TaqMan probes and PCR primers directed to 84 human drug transporter genes (29 ABC transporters, 46 SLC transporters and 9 others) and 12 candidate endogenous control genes. PCR runs were carried in triplicate out on a Viia7 PCR instrument (Life Technologies). For all analysis, the maximum allowable cycle threshold (Ct) value was fixed at 35. Baseline transcript levels were calculated as ratio between the mean Ct value of the three most stable endogenous control genes (HPRT1, RPLP0, UBC) and the Ct value for the target gene. Stability of endogenous control genes was determined on the basis of the lowest SD score which is an indicator of consistent expression across all samples and is calculated using geometric averaging [21]. For clarity, baseline mRNA expression levels of unstimulated tissues and cell lines were categorized as low, intermediate and high and corresponded to ratio value ranges of 0.65–0.80, 0.81–1 and > 1, respectively. Relative transcripts levels in drug-stimulated cells were determined using the comparative Ct method (ΔΔCt method) [22]. Fold change values (fc) were considered as follows: fc<0.5 = downregulated, 0.5<fc>2 stable, 2<fc>4 = low expression, 4<fc>10 moderate expression, fc>10 = high expression. All analyses were conducted using the DataAssist software (Life Technologies).

### Statistical Analysis

Similarities of gene expression between tissue and cell lines groups was assessed using Pearson’s product moment correlation (r) with the significance level for correlation set at P≤ 0.05. To assess the difference between drug-treated versus untreated cells at different time points, a fold change cut-off of 3 was considered to perform statistical analysis by one-way ANOVA with Dunnett’s post hoc test. All values are represented as means ± SD (n = 3). Differences were considered statistically significant when P≤ 0.05. Statistical analyses were conducted using the GraphPad Prism 6 software.

## Results

### Drug concentrations for induction assays

Viability of cell lines in presence of ARV drugs at concentrations as high as those used in the context of *in vivo* studies was assessed. To avoid drug-induced antiproliferative effects on cell lines, we elected to stimulate cells with drug concentrations which showed inhibition of cell proliferation not greater than 20%. Cell line incubation with tenofovir showed no reduction in cell viability at concentrations up to 1 mM (data not shown). Cell treatment with darunavir and dapivirine at concentrations of 10 μM and 250 μM respectively showed cell vitality within the acceptable range (90±10%), while higher concentrations resulted in marked reduction in cell vitality (data not shown).

### Expression of drug transporters in cervicovaginal tissue and cell lines

Expression of ABC and SLC transporters in cervicovaginal cell lines and mucosal explants is summarized in Tables 1 and 2, respectively. Expression levels varied across the cervical region with both ABC and SLC transporters consistently expressed at higher levels in ectocervix compared to endocervix. Cervical explants were qualitatively richer in both ABC and SLC transporters compared to vaginal explants. The following ABC transporters implicated in transport of ARVs were all expressed in ectocervix: P-gp, BCRP, MRP1, MRP2, MRP3, MRP4, MRP5, MRP6 and MRP7 while MRP2, MRP3, MRP4 and MRP6 were not expressed in vaginal samples. Likewise, the following SLC transporters implicated in transport of ARVs were all expressed in ectocervix: OCT1, OCT3, CNT1, CNT2, CNT3, ENT1, ENT2, OATP8, OAPT2A1, OATP2B1, OATPD and OATPE while none of the CNT transporters, OCT1, OCT2, ENT2 and OATP8 were expressed in vaginal samples.

**Table 1:** Expression of genes for ABC drug transporters in cervicovaginal cell lines and tissues.

ABC transporters^a^	Gene expression level^b^							
	*Cell lines*					*Tissues*		
	HEC-1A	VK/E6E7	HeLa	End/E6E7	Ect/E6E7	Ectocervix	Endocervix	Vagina
ABCA1	-	++	+	++	+	++	+	++
ABCA12	+	+	-	+	+	++	-	-
ABCA13	+	+	-	+	+	+	-	-
ABCA2	++	+	++	+	+	++	+	++
ABCA3	++	+	++	+	++	+	+	++
ABCA4	+	-	++	-	-	+	+	-
ABCA9	+	-	-	-	-	++	+	++
P-gp	+	-	+	+	+	++	+	++
ABCB6	+	+	++	+	+	+	+	++
MRP1	++	++	++	++	++	++	+	++
MRP7	+	+	+	+	+	+	+	++
MRP8	+	+	-	+	+	-	-	-
MRP2	+	+	+	+	+	+	-	-
MRP3	++	+	+	+	+	+	+	-
MRP4	++	+	++	+	+	+	+	-
MRP5	++	+	++	++	++	++	++	++
MRP6	+	+	+	+	+	+	-	-
ABCD1	+	+	+	+	+	+	-	++
ABCD3	++	++	++	++	++	++	++	++
ABCD4	++	++	++	++	++	++	+	++
ABCF1	++	++	++	++	++	++	++	++
BCRP	-	+	+	+	+	++	+	++
TAP1	++	++	+	++	++	++	+	++
TAP2	++	++	++	++	++	++	+	++

^a^ Five ABC transporter genes were not expressed in any of the cell lines or tissues: BSEP, MDR2, ABCB5, MRP9 and ABCG9.

^b^Data were calculated as Ct(control gene)/Ct(target gene). Values obtained correspond to ratio ranges of <0.65 indicated as—; 0.65–0.80 as +, 0.81–1 as ++ and >1 as +++. Data are the result of the mean of three biological replicates.

**Table 2:** Expression of genes for SLC drug transporters in cervicovaginal cell lines and tissues.

SLC transporters^a^	Gene expression level^b^							
	*Cell lines*					*Tissues*		
	HEC-1A	VK2/E6E7	HeLa	End1/E6E7	Ect1/E6E7	Ectocervix	Endocervix	Vagina
PEPT1	-	+	-	+	-	+	+	++
PEPT2	+	+	+	+	+	+	++	-
MCT1	+	++	++	++	++	++	+	++
MCT7	+	+	-	+	+	+	-	++
MCT3	++	++	++	++	++	++	++	++
SLC19A1	++	+	+	++	+	+	-	++
THTR1	++	+	++	+	+	++	+	++
THTR2	-	+	-	+	+	+	-	-
OCT1	+	+	+	+	+	+	-	-
OCT2	+	-	-	-	-	-	-	-
OCT3	-	+	++	+	+	++	+	++
CITRIN	++	++	++	++	++	++	+	++
CNT1	+	+	-	-	-	+	-	-
CNT2	-	+	-	-	-	++	-	-
CNT3	-	+	-	+	+	+	+	-
ENT1	++	++	++	++	++	++	++	++
ENT2	++	+	+	+	++	+	-	-
GLUT1	++	++	++	++	++	++	++	++
GLUT3	+	+	+	+	+	++	+	++
SLC31A1	++	++	++	++	++	++	++	++
SLC38A2	++	++	++	++	++	++	++	++
SLC38A5	+	++	++	++	++	++	+	++
NBAT	+	+	-	-	-	+	-	-
SLC3A2	++	++	+++	+++	++	++	++	++
SGTL1	+	+	-	-	+	++	++	++
SGLT3	+	-	-	-	-	+	+	++
SLC7A11	++	+	++	++	+	+	-	-
SLC7A5	++	++	++	+++	++	++	+	++
SLC7A6	+	++	++	+	+	+	+	-
SLC7A7	+	+	+	-	-	+	-	-
SLC7A8	+	++	+	++	++	++	++	++
SLC7A9	+	+	+	+	-	+	+	-
OATPC	+	-	+	-	-	-	-	-
OATP8	+	-	++	-	-	+	+	-
OATP2A1	+	++	++	-	+	++	++	++
OATP2B1	+	-	-	-	-	+	+	++
OATPD	+	+	+	++	+	+	+	++
OATPE	++	+	++	+	+	+	+	++

^a^ Eight SLC transporter genes were not expressed in any of the cell lines or tissues: SLC10A2, NCTP, OAT1, OAT2, OAT3, OAT4, GLUT2 and OATP.

^b^Data were calculated as Ct(control gene)/Ct(target gene). Values obtained correspond to ratio ranges of < 0.65 indicated as—; 0.65–0.80 as +, 0.81–1 as ++ and > 1 as +++. Data are the result of the mean of three biological replicates.

There was good association of ABC transporters expression between HeLa, Ect1/E6E7 and End1/E6E7 cells and cervical explants (r = 0.63–0.65), although P-gp and BCRP were expressed at lower levels in cell lines compared to ectocervix. HEC-1A cells also showed good association with cervical explants with higher expression of MRP3 and MRP4 compared to explants, but no BCRP expression. VK2/E6E7 cells showed low expression of all MRP transporters (except MRP1), BCRP and absence of P-gp expression. Expression of OCT and ENT influx transporters in ectocervical tissue matched expression in HeLa cells, while all CNT transporters were absent in this cell line, as was the case for vaginal tissue. VK2/E6E6 and HeLa cells expressed OATP2A1 at similar levels compared to tissues, while the other cell lines showed lower or no expression of this transporter. VK2/E6E7 cells showed additional similarities with vaginal tissue in expression of members of the SLCO family, with absence of OATP8 and OATPC and expression of OATPD and OAPTE, although at lower levels compared to tissue. OATP2B1 was absent in VK2/E6E6, and only expressed at low levels in tissues and HEC1-A cells. Overall expression of other SLC transporters in HEC-1A correlated poorly with cervical explants, with no expression of OCT3, CNT2 and CNT3 in HEC-1A.

### Effect of darunavir on expression of drug transporters in cervicovaginal cell lines

**Fig 1:**
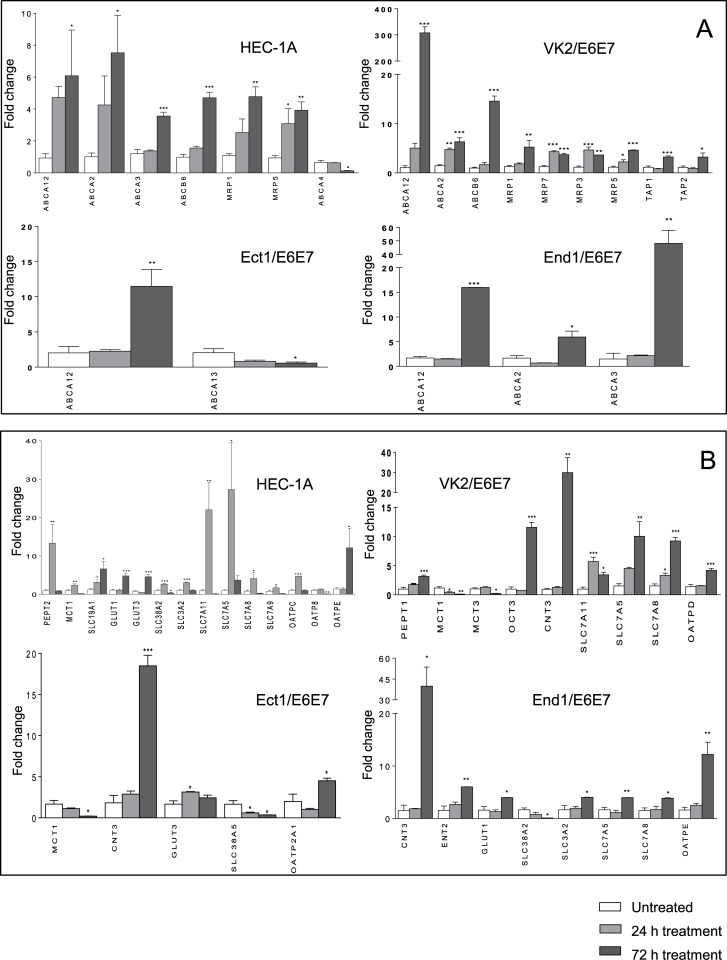
Drug transporters gene expression in darunavir-treated cell lines. Cell lines were treated with 250 μM darunavir for 24 and 72 hours. Levels of total mRNA were measured by Real Time PCR. Relative quantitation was determined using the comparative Ct method with data normalized to three housekeeping genes (HPRT1, RPLP0, UBC) and calibrated to the average ΔCt of untreated controls (fold induction = 2^-ΔΔCt^). A) ABC transporters gene expression in darunavir-treated HEC-1A, VK2/E6E7, Ect-1/E6E7 and End-1/E6E7 cell lines. B) SLC transporters gene expression in darunavir-treated HEC-1A, VK2/E6E7, Ect-1/E6E7 and End-1/E6E7 cell lines. Values are represented as mean ± SD of at least three independent experiments. A fold change cut-off of 3 was used for statistical analysis by one-way ANOVA and Dunnet’s post test. Only genes which showed statistically significant changes of expression are reported (*P* ≤ 0.05).

Differences of gene expression of ABC and SLC transporters in darunavir-treated cell lines compared to DMSO-treated cells that reached statistical significance are shown in Fig 1. All ABC and SLC transporters genes which expression was affected by darunavir in at least one cell line are shown in S1 Table. Unless otherwise stated all expression changes outlined below were observed after cell line stimulation for 72 hours. No significant differences in levels of gene upregulation or downregulation were observed in cells treated for 168 hours compared to 72 hours treatment (data not shown). Stimulation of cervicovaginal cell lines with darunavir (250 μM) upregulated MRP1, MRP5 and MRP7 (up to 5-fold increase) in VK2/E6E7 and HEC-1A, although upregulation of MRP7 in the latter was not statistically significant. MRP 3 was significantly upregulated only in VK2/E6E7 (P < 0.0001). Darunavir showed no significant effect on expression of ABC transporters P-gp, MRP2 and BCRP in any of the cell lines. Expression of SLC transporter CNT3 was significantly induced by >10-fold in VK2/E6E7, Ect1/E6E7 and End1/E6E7. OCT3 was also markedly induced in VK2/E6E7 (P < 0.0001) and ENT2 in End1/E6E7 cells (P < 0.001) but not in the other cell lines. As shown in Fig 1, darunavir did not upregulate CNT, OCT and ENT transporters in HEC-1A while changes in expression of SLCO transporters were seen in this cell line with OATPE upregulated by 12-fold after 72 hours (P < 0.05), OATPC upregulated by 4.7-fold after 24 hours (P < 0.0001) and downregulation of OAPT8 (P < 0.001) at 72 hours. Expression of OATPE in End1/E6E7 was also strongly induced by darunavir (12-fold increase, P < 0.001). OATP2A1 and OATPD were modestly upregulated in Ect1/E6E7 and VK2/E6E7, respectively. The effect of darunavir on efflux and uptake transporters not involved in transport of ARV drugs is also shown in Fig 1. No expression changes were seen in cells stimulated with 0.1% DMSO alone (data not shown).

### Effect of dapivirine on expression of drug transporters in cervicovaginal cell lines

**Fig 2:**
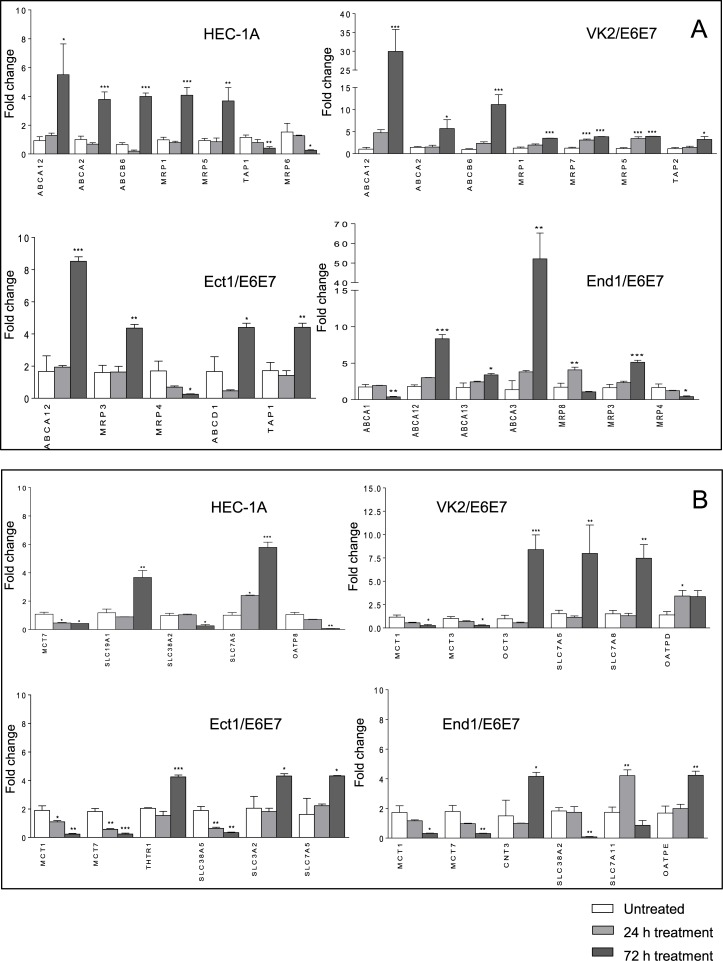
Drug transporters gene expression in dapivirine-treated cell lines. Cells were treated with 10 μM dapivirine for 24 and 72 hours. Levels of total mRNA were measured by Real Time PCR. Relative quantitation was determined using the comparative Ct method with data normalized to three housekeeping genes (HPRT1, RPLP0, UBC) and calibrated to the average ΔCt of untreated controls (fold induction = 2^-ΔΔCt^). A) ABC transporters gene expression in dapivirine-treated HEC-1A, VK2/E6E7, Ect-1/E6E7 and End-1/E6E7 cell lines. B) SLC transporters gene expression in dapivirine-treated HEC-1A, VK2/E6E7, Ect-1/E6E7 and End-1/E6E7 cell lines. Values are represented as mean ± SD of at least three independent experiments. A fold change cut-off of 3 was used for statistical analysis by one-way ANOVA and Dunnet’s post test. Only genes which showed statistically significant changes of expression are reported (P ≤ 0.05).

Differences of gene expression of ABC and SLC transporters in dapivirine-treated cell lines compared to DMSO-treated cells that reached statistical significance are shown in Fig 2. All ABC and SLC transporters genes which expression was affected by dapivirine in at least one cell line are shown in S2 Table. Unless otherwise stated all expression changes outlined below were observed after cell line stimulation for 72 hours. No significant differences in levels of gene upregulation or downregulation were observed in cells treated for 168 hours compared to 72 hours treatment (data not shown). Dapivirine (10 μM) significantly upregulated efflux transporters MRP1 and MRP5 in VK2/E6E7 and HEC-1A, and downregulated MRP4 consistently across HEC-1A, Ect1/E6E7, End1/E6E7, but not at statistically significant levels in HEC-1A. Upregulation of MRP3 was also seen in Ect1/E6E7 and End1/E6E7 (5-fold, P < 0.0001 and 4-fold, P < 0.001 respectively). MRP7 was significantly upregulated in VK2/E6E7 and MRP6 was downregulated in HEC-1A. Dapivirine showed no effect on expression of P-gp, MRP2 and BCRP in any of the cell lines. SLC transporters OCT3 and CNT3 were upregulated by dapivirine in VK2/E6E7 (8-fold, P < 0.0001) and End1/E6E7 (4-fold, P < 0.05), respectively. Dapivirine showed no significant effect on expression of ENT, CNT and OCT transporters in HEC-1A and Ect1/E6E7 cell lines. Expression of SLCO transporter OATPD was weakly but significantly induced in VK2/E6E7 only at 24 hours (3-fold, P < 0.05). Other effects on SLCO transporters include downregulation of OATP8 in HEC-1A (14-fold, P < 0.001) and upregulation of OATPE in End1/E6E7 (4-fold, P < 0.001). The effect of dapivirine on efflux and uptake transporters not involved in transport of ARV drugs is also shown in Fig 2. No expression changes were seen in HeLa cells.

### Effect of tenofovir on expression of drug transporters in cervicovaginal cell lines

**Fig 3:**
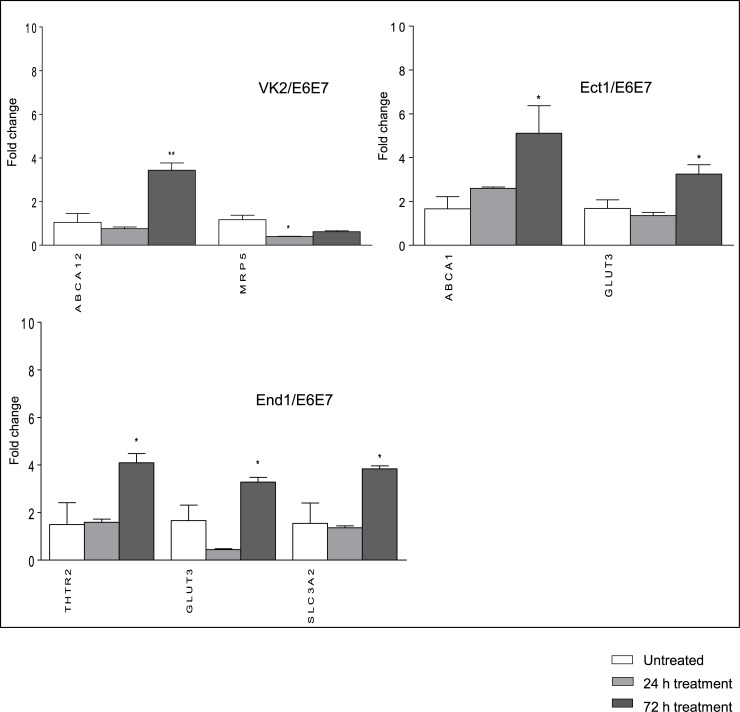
Drug transporters gene expression in tenofovir-treated cell lines. Cells were treated with 1000 μM tenofovir for 24 and 72 hours. Levels of total mRNA were measured by Real Time PCR. Relative quantitation was determined using the comparative Ct method with data normalized to three housekeeping genes (HPRT1, RPLP0, UBC) and calibrated to the average ΔCt of untreated controls (fold induction = 2^-ΔΔCt^). ABC and SLC transporters gene expression in tenofovir-treated VK2/E6E7, Ect-1/E6E7 and End-1/E6E7 cell lines. Values are represented as mean ± SD of at least three independent experiments. A fold change cut-off of 3 was used for statistical analysis by one-way ANOVA and Dunnet’s post test. Only genes which showed statistically significant changes of expression are reported (P ≤ 0.05).

Differences of gene expression of ABC and SLC transporters in tenofovir-treated cell lines compared to DMSO-treated cells that reached statistical significance are shown in Fig 3. All ABC and SLC transporters genes which expression was affected by tenofovir in at least one cell line are shown in S3 Table. Stimulation with tenofovir (1 mM) mostly resulted in non-statistically significant expression changes which were limited to a few drug transporters. Downregulation of MRP5 in VK2/E6E7 at 24 hours was the only expression change that reached statistical significance among transporters relevant to ARV drugs (3-fold, P < 0.05). As shown in Fig 3 among transporters not relevant for ARV the following were significantly upregulated: ABCA12 in VK2/E6E7; THTR2, GLUT3 and SLC3A2 in End1/E6E7 cells; ABCA1 and GLUT3 in Ect1/E6E7.

## Discussion

Characterisation of expression of drug transporters in the cell lines here analysed and comparison with expression in cervicovaginal tissue provides insight into their suitability as surrogates for cervicovaginal tissue in transport kinetics studies of candidate microbicides. Expression analyses of drug transporters in cervicovaginal tissue and cell lines include all transporters implicated in transport of protease and reverse transcriptase inhibitors. Therefore, the study provides a significant addition to the current literature to inform which transporters should be represented in cell line-based permeability studies for assessment of microbicide formulations. Our findings show a good correlation of expression of ABC transporters between HeLa, Ect1/E6E7, End1/E6E7 cells and cervical tissues, except for lower expression of BCRP and P-gp in cell lines compared to ectocervical tissue. Subject to confirming functionality and localization of the detected efflux transporters, HeLa, Ect1/E6E7 and End1/E6E7 engineered to over-expressed BCRP and P-gp could serve as ectocervical tissue surrogates in transport kinetics studies for evaluation of efflux of protease and reverse transcriptase inhibitors. Among influx transporters involved in transport of nucleotide analogues, expression of ENT influx transporters in ectocervical tissue matched expression in HeLa cells, while all CNT transporters were absent in this cell line, in this respect displaying stronger similarity with vaginal tissue. Expression of OCT involved in transport of protease and reverse transcriptase inhibitors was also best represented in HeLa cells. Expression of SLCO transporters in vaginal tissues was best reflected in VK2/E6E7 cells except for absence of OATP2B1 and lower expression of OATPD and OATPE compared to vaginal tissue. Our data suggest that cell line choice for evaluation of drug influx should be informed by the class of drugs tested. As above, subject to confirming functionality and localization of the detected influx transporters, HeLa cells may be a good ectocervical tissue surrogate for testing uptake of nucleotide analogue reverse transcriptase inhibitors and VK2/E6E7 a more suitable vaginal tissue surrogate for testing uptake of protease inhibitors. The known ability of HEC-1A cells to form tight junctions [23] makes this cell line a more suitable candidate for monolayer-based transport kinetics studies [24]. However, differences in drug transporter expression of HEC-1A compared to cervicovaginal tissue should be considered when developing such assays, namely higher expression of MRP transporters, no expression of BCRP as well as differences in expression of SLC transporters.

Our findings in relation to drug transporter expression in cervicovaginal tissue are only partially confirmatory of recent reports [12,25]. Inter-patient expression profiles of both ABC and SLC transporters were consistent across all women recruited whereas qualitative differential expression was observed between cervical and vaginal samples. Cervical explants were generally richer in both ABC and SLC transporters compared to vaginal explants. Quantitative expression levels across the cervical region varied with both ABC and SLC transporters consistently expressed at higher levels in ectocervix compared to endocervix. Expression of OAT transporters was not observed in either cervicovaginal tissue or cell lines. A further report confirmed the absence of OAT1 and OAT3 in cervicovaginal explants but showed expression of OAT2 [12] which we did not detect. Expression of OAT transporters, which we were not able to confirm, may have implications in mucosal disposition of topically applied nucleoside analogues [14]. Additional qualitative and quantitative differences in expression of SLC transporters include equal levels of OCT3 in cervix and vagina, no expression of OCT2 in any tissue samples, expression of ENT2 and CNT transporters in ectocervix but not vagina. Expression of OCT transporters may have implications in mucosal disposition of both nucleoside analogues and protease inhibitors whereas CNTs and ENTs have only been implicated in transport of nucleoside analogues. We confirmed expression of OATPD and OATPE in all tissue samples and observed expression of additional SLCO transporters implicated in transport of several protease inhibitors [14]. In agreement with Zhou et al, we showed no expression of MRP2, MRP3 and MRP6 in vagina, although low levels of these MRP transporters were detected in ectocervix. Of note, a recent report [26] showed expression of MRP2 in both vagina and endocervix. The gene expression profile for drug transporters in mucosal CD4+ T cells purified from explants was also analysed (data not shown). Expression in CD4+ T cells did not correlate with that of wholly-processed cervicovaginal explants reported in this study. This suggests that gene expression findings shown here are not representative of drug transporters expressed in mucosal CD4+ T cells.

Cell line stimulation experiments showed a modulatory effect by ARV drugs on expression of various transporters implicated in transport of nucleotide analogue reverse transcriptase inhibitors, non-nucleoside analogue reverse transcriptase inhibitors and protease inhibitors. We aimed to test the effect of dapivirine, darunavir and tenofovir at concentrations relevant for clinical trials while minded of potential cytotoxic effects on the cell lines. Vaginally-applied tenofovir gel showed protection at a concentration of 1%. Dapivirine vaginal ring (200 mg) is currently in clinical trials individually (The Ring Study, International Partnership for Microbicides; ASPIRE, Microbicide Trials Network) [27,28] and has been tested in combination with darunavir (300 mg) in non-human primate models [29]. The drug concentrations used for stimulation of cell lines in this study were the highest concentrations which showed no cytotoxicity in XTT assays and maintained solubility in culture medium. No expression changes of drug transporters by dapivirine, darunavir and tenofovir were observed at concentrations lower than 10 μM, 250 μM and 1 mM, respectively. Equally no expression changes of drug transporters were observed in cells stimulated with 0.1% DMSO alone. Rifampicin and cisplatin, chosen as potential reference compounds in view of their inducing effect on P-gp and MRP transporters in liver, kidney and intestinal cell lines [30], displayed little or no effect on expression of efflux transporters in cervico-vaginal cell lines (data not shown) and were deemed not suitable positive controls in our study. Upon stimulation with drugs, most expression changes that reached statistical significance were seen after stimulation for 72 hours as opposed to 24 hours in line with previous reports [20], whereas stimulation for 168 hours showed no significant differences in gene upregulation or downregulation compared to 72 hours treatment. Expression changes of transporters in drug-treated cells mostly differed across cell lines tested, although darunavir-induced changes were more consistent across different cell lines compared to dapivirine, particularly for uptake transporters. Similar trends in modulatory effect induced by darunavir and dapivirine were seen across cell lines for a number of transporters involved in transport of ARV drugs as summarized in S1 and S2 Tables, although statistical significance of these expression changes was often limited to 1 or 2 cell lines. In this respect differential effects of antiretrovirals on drug transporter expression and activity in cell lines derived from similar tissues have been often reported in literature [20,31,32]. Despite good correlation of drug transporter expression in HeLa cells with ectocervical tissue, these cells may not be suitable to test transport of drugs analysed in this study which showed no significant effect on expression of drug transporters in this cell line.

Darunavir and dapivirine significantly upregulated MRP1 and MRP5 in VK2/E6E7 and HEC-1A. MRP1 has been repeatedly confirmed to be substrate for protease inhibitors [33,34] while MRP5 has been shown to be substrate for nucleotide analogue reverse transcriptase inhibitors [35] but not non-nucleoside analogue reverse transcriptase inhibitors and protease inhibitors as yet. Tenofovir substrate MRP4 [36] was consistently downregulated by dapivirine but not darunavir in HEC-1A, Ect1/E6E7 and End1/E6E7, reaching statistical significance in both Ect1/E6E7 and End1/E6E7. We observed no significant effect by any of the drugs on expression of MRP2, BCRP and P-gp widely implicated in efflux of several classes of ARVs [14,37,38]. Upregulation of MRP2, involved in efflux of some nucleotide analogues including tenofovir [38], by dapivirine was observed in HEC-1A and VK2/E6E7 but not at statistically significant levels. Darunavir was previously shown to induce P-gp expression in lymphocytes [18] and LS180 intestinal cells [20] but not in Caco-2 intestinal cells [20]. The latter study also showed no effect by darunavir on expression of BCRP and MRP2 in intestinal cells. Upregulation of transporters involved in uptake of ARV drugs by dapivirine was significant in VK2/E6E7 and End1/E6E7 but not in the cervical cell lines. Influx transporters upregulated by darunavir across different cell lines included OCT, ENT and CNT transporters involved in uptake of tenofovir [13] as well as members of the SLCO transporters involved in transport of protease inhibitors themselves.

This study along with functional characterisation of drug uptake and efflux transporters in cervicovaginal tissue informs development of microbicide combinations [39,40] which could be modified by beneficial drug-drug interactions or by including selective inhibitors or inducers of drug transporters to optimize drug concentrations at target tissue sites. Our findings may suggest that in a vaginally-delivered tenofovir-darunavir microbicide combination the distribution of individual drugs at subepithelial CD4+ T cells could be affected by expression changes of drug transporters. The net transfer of drug across the epithelium will depend on the cell surface distribution and functional activity of transporters which expression is induced or suppressed. Specifically, concurrent upregulation of MRP1, MRP5, MRP7, CNT3 and OCT3 by darunavir may result in increased transfer across the epithelium of substrates for these transporters such as tenofovir, if MRP transporters are expressed on the baso-lateral surface and influx transporters on the apical surface of cervicovaginal cells as reported in other tissues [41]. Darunavir has been shown to increase apical-to-basolateral tenofovir disoproxil fumarate permeation in Caco-2 cells [42]. Conversely transport across the epithelium may be restricted if the upregulated MRP transporters were expressed on the apical surface. In this respect, tenofovir was shown to induce intracellular accumulation of an MRP substrate in kidney cell lines [16]. Stimulation with tenofovir itself resulted in low but statistically significant downregulation of MRP5 only at 24 hours. A previous study has shown a downregulatory effect of tenofovir on expression of MRP5 in peripheral blood mononuclear cells [43]. However this effect may not impact long term disposition of tenofovir in a vaginal microbicide in view of the relatively transient effect on expression in VK2/E6E7 vaginal cell line. On the other hand the observed upregulation of SLCO transporters by darunavir, may augment intracellular accumulation of darunavir itself as previously suggested for intestinal cells [20] and result in decreased distribution of darunavir to CD4+ T cells. Interestingly, in HEC-1A we observed concurrent upregulation and downregulation of SLCO transporters which may ultimately diminish the net effect of these transporters on uptake of darunavir.

Similar effects on distribution of tenovofir at target sites may also result from a vaginally-delivered tenofovir-dapivirine microbicide combination, in view of similar effects of dapivirine on expression of MRP1, MRP5, OCT3 and CNT3. Upregulation of CNT3 by dapivirine was only observed in End1/E6E7 and was markedly lower compared to darunavir. Additional effects on net transport of tenofovir across the epithelial barrier may also derive from downregulation of apical efflux transporter MRP4 which was not observed in darunavir-stimulated cells. Upregulation of MRP3, consistently observed in dapivirine-stimulated cells may be counteracted by the inhibitory effect of tenofovir on this transporter [16]. The effect of darunavir on expression of SLCO transporters described above may be enhanced by combination with dapivirine in view of the upregulatory effect of dapivirine on OATPE. Drug transporters involved in transport of dapivirine have not been specifically characterized yet. It is therefore difficult to speculate on the potential effect of expression changes of drug transporters on distribution of vaginally-delivered dapivirine.

Investigation of protein expression and cell surface distribution along with functional analyses of drug transporters will clarify the implications of the drug-induced gene expression changes here described. Functional analyses will include cell line-based transport kinetics studies informed of our findings of drug transporter expression in cell lines. Expression changes of MRP mRNA levels were previously shown to correlate with changes in corresponding protein or altered function [20,44]. Future *in vivo* pharmacokinetics studies will investigate whether combinations of darunavir and/or dapivirine with tenofovir affect retention of individual drugs at the cervicovaginal mucosae. The effect of ARVs on expression of drug transporters should also be interpreted in conjunction with the potential effect of HIV-1 infection itself on drug transporter expression in cervicovaginal tissue which has yet to be determined.

## Supporting Information

S1 Table Effect of darunavir on expression of drug transporters in cervico-vaginal cell lines. (DOCX)

S2 Table Effect of dapivirine on expression of drug transporters in cervico-vaginal cell lines. (DOCX)

S3 Table Effect of tenofovir on expression of drug transporters in cervico-vaginal cell lines. (DOCX)
