# Supplementary material for: Expression of Genes for Drug Transporters in the Human Female Genital Tract and Modulatory Effect of Antiretroviral Drugs
Source: PLoS One. 2015 Jun 23;10(6):e0131405. doi: 10.1371/journal.pone.0131405 (PMC4477895; doi:10.1371/journal.pone.0131405)
Supplement: S2 Table — (DOCX) [file pone.0131405.s002.docx]

**S2 Table. Effect of dapivirine on expression of drug transporters in cervico-vaginal cell lines.**

| Drug transporters^a^ | Gene expression level^b^ | | | | | | | |
| --- | --- | --- | --- | --- | --- | --- | --- | --- |
|  | HEC-1A | | VK2/E6E7 | | Ect1/E6E7 | | End1/E6E7 | |
|  | 24 h | 72 h | 24 h | 72 h | 24 h | 72 h | 24 h | 72 h |
| ABCA1 |  |  |  |  |  |  |  | ↓ |
| ABCA12 |  | ↑↑ | ↑↑ | ↑↑↑ |  | ↑↑ | ↑ | ↑↑ |
| ABCA13 |  |  |  |  |  |  | ↑ | ↑ |
| ABCA2 |  | ↑ | ↑ | ↑↑ |  |  |  | ↑ |
| ABCA3 |  |  | ↑↑ | ↑↑ |  | ↑ | ↑ | ↑↑↑ |
| ABCA4 | ↓ | ↓ |  |  |  |  |  |  |
| ABCB6 | ↓ | ↑ | ↑ | ↑↑↑ |  | ↑ |  | ↑ |
| MRP1 |  | ↑↑ | ↑ | ↑ |  | ↑ |  | ↑ |
| MRP2 |  | ↑ | ↑ | ↑ |  |  |  |  |
| MRP7 |  | ↑ | ↑ | ↑ |  |  |  |  |
| MRP3 |  |  |  | ↑↑ |  | ↑↑ | ↑ | ↑↑ |
| MRP4 | ↓ | ↓ | ↓ |  |  | ↓ |  | ↓ |
| MRP5 |  | ↑ | ↑ | ↑ |  | ↑ |  | ↑ |
| MRP6 |  | ↓ |  |  |  |  |  |  |
| MRP8 |  |  |  |  |  |  | ↑↑ |  |
| ABCD1 |  |  | ↑ |  |  | ↑↑ |  | ↑ |
| TAP1 |  | ↓ | ↑ | ↑↑ |  | ↑↑ |  | ↑ |
| TAP2 |  | ↑ |  | ↑ |  |  |  |  |
| PEPT2 |  |  |  |  |  | ↑ |  | ↑↑ |
| MCT1 |  | ↓ |  | ↓ |  | ↓ |  | ↓ |
| MCT7 | ↓ | ↓ |  |  |  | ↓ |  | ↓ |
| MCT3 |  | ↓ |  | ↓ |  |  |  |  |
| THTR1 |  |  | ↑ |  |  | ↑↑ |  | ↑ |
| THTR2 |  |  | ↓ | ↓ |  |  |  |  |
| ENT1 |  |  |  |  |  | ↓ |  |  |
| ENT2 |  |  | ↑ |  |  | ↑ | ↑ | ↑ |
| CNT3 |  |  |  | ↑ | ↑ | ↑ |  | ↑ |
| GLUT1 |  |  | ↑ |  |  | ↑ |  | ↑↑ |
| GLUT3 |  |  | ↑ |  | ↑ |  |  |  |
| SLC38A2 |  | ↓ |  |  |  |  |  | ↓ |
| SLC38A5 |  |  |  |  |  | ↓ |  |  |
| OCT1 |  |  |  | ↑ |  | ↑ | ↑ | ↑↑ |
| OCT3 |  |  |  | ↑↑ |  | ↑ |  | ↑ |
| NBAT | ↓ | ↓ | ↓ |  |  |  |  |  |
| SLC3A2 | ↑ | ↑ | ↑ |  |  | ↑↑ |  | ↑ |
| SLC7A11 | ↑ | ↓ | ↑↑↑ |  | ↑↑ | ↑ | ↑↑ |  |
| SLC7A5 | ↑ | ↑↑ |  | ↑↑ | ↑ | ↑↑ |  | ↑ |
| SLC7A7 |  |  |  |  |  | ↑ | ↑ | ↑↑ |
| SLC7A8 |  |  |  | ↑↑ |  | ↑ |  | ↑ |
| SLC7A9 |  |  | ↑ |  |  |  |  | ↑↑ |
| OATP8 |  | ↓ |  |  |  |  |  |  |
| OATP2A1 |  |  | ↓ | ↓ |  |  |  |  |
| OATP2B1 | ↓ | ↓ |  |  |  |  |  |  |
| OATPD |  |  | ↑ | ↑ |  | ↑ |  |  |
| OATPE |  | ↑ |  |  | ↑ | ↑ | ↑ | ↑↑ |

^a^ Drug transporters with fold change (fc) values <0.5 and >2 in at least one cell line.

^b^ Fold change (fc) values are represented as follows: fc<0.5 as ↓, 2<fc>4 as ↑ , 4<fc>10 as ↑↑, fc>10 as ↑↑↑. Blank cells indicate no fold changes (0.5<fc>2). Data are the result of the mean of three biological replicates.
